# Supplementary material for: Effect of music therapy on behavioral and physiological neonatal outcomes: A systematic review and dose-response meta-analysis
Source: PLoS One. 2025 Jan 8;20(1):e0316674. doi: 10.1371/journal.pone.0316674 (PMC11709260; doi:10.1371/journal.pone.0316674)
Supplement: S1 Table — (DOCX) [file pone.0316674.s004.docx]

**Supplementary table 1:** Characteristics of the excluded studies

| Row | Study | Reason for exclusion |
| --- | --- | --- |
| 1 | Abadian 2020 | The research sample was not pre-term neonates, but their mothers. |
| 2 | Adén 2014 | An editorial article rather than original article. |
| 3 | Aikman 1997 | A congress paper without abstract. |
| 4 | Aita 2018 | Trial protocol rather than original article. |
| 5 | Aita 2021 | A meta-analysis article rather than original article. |
| 6 | Ak 2015 | The research sample was not pre-term neonates, but their mothers. |
| 7 | Alabbasi 2023 | A systematic review rather than original article. |
| 8 | Anderson 1986 | It is not a randomized clinical trial about music therapy. |
| 9 | Anderson 2018 | A commentary article rather than original article. |
| 10 | Anuntaseree 2020 | It is not a randomized clinical trial about music therapy. |
| 11 | Arnon 2006 | There was no control group data. |
| 12 | Arnon 2022 | An case control study rather than randomized clinical trial. |
| 13 | Ashley 2010 | A research letter rather than original article. |
| 14 | Ashton 2023 | It is not a randomized clinical trial about music therapy. |
| 15 | Asirvatham 2006 | Randomization was not done, and the outcome of interest was not physiological or behavioral. |
| 16 | Aucott 2002 | Randomization was not done, and the outcome of interest was not physiological or behavioral. |
| 17 | Aydin 2012 | Randomization was not done. |
| 18 | Barcos-Munoz 2024 | The outcomes and effect sizes reported were inconsistent with the present study. |
| 19 | Barnard 1983 | It is not a randomized clinical trial about music therapy. |
| 20 | Barreto 2006 | Randomization was not done, and the outcome of interest was not physiological or behavioral. |
| 21 | Bastani 2017 | There was no music therapy arm. |
| 22 | Baud 2022 | It is not a randomized clinical trial about music therapy. |
| 23 | Bauer 2021 | A cross-sectional study rather than randomized clinical trial. |
| 24 | Bauer-Rusek 2024 | The outcomes and effect sizes reported were inconsistent with the present study. |
| 25 | Becker 2015 | A systematic review rather than original article. |
| 26 | Bellido 1995 | There was no music therapy arm. |
| 27 | Bellieni 2003 | There was no music therapy arm. |
| 28 | Benoit 2017 | A systematic review rather than original article. |
| 29 | Bergomi 2014 | Effect sizes are reported as figures, not numerical values. |
| 30 | Bernadou-Debrulle 2021 | A systematic review rather than original article. |
| 31 | Bertsch 2020 | It is not a randomized clinical trial. |
| 32 | Best 2018 | A meta-analysis article rather than original article. |
| 33 | Bhat 2018 | An editorial article rather than original article. |
| 34 | Bhatia 2006 | There was no music therapy arm. |
| 35 | Bieleninik 2016 | A meta-analysis article rather than original article. |
| 36 | Bieleninik 2020 | A congress abstract rather than original article. |
| 37 | Bieleninik 2021 | A narrative review rather than original article. |
| 38 | Birkeland 1993 | It is not a randomized clinical trial about music therapy. |
| 39 | Blanco-Dávila 2001 | It is not a randomized clinical trial about music therapy. |
| 40 | Bonjorn 2020 | Trial protocol rather than original article. |
| 41 | Bovo 2015 | A case-series study rather than original article. |
| 42 | Brand 2012 | There was no music therapy arm. |
| 43 | Bremmer 2003 | It is not a randomized clinical trial. |
| 44 | Breuillard 2015 | It is not a randomized clinical trial about music therapy. |
| 45 | Brown 2009 | It is not a randomized clinical trial. |
| 46 | Burke 1995 | A case-series study rather than original article. |
| 47 | Butt 2000 | It is not a randomized clinical trial. |
| 48 | Caine 1991 | A systematic review rather than original article. |
| 49 | Calabro 2003 | Randomization was not done. |
| 50 | Campbell-Yeo 2012 | Effect sizes are reported as figures, not numerical values. |
| 51 | Cardoso 2014 | There was no music therapy arm. |
| 52 | Carvalho 2019 | It is not a randomized clinical trial. |
| 53 | Cassidy 1999 | It is not a randomized clinical trial. |
| 54 | Cassidy 2009 | It is not a randomized clinical trial. |
| 55 | Cavaiuolo 2015 | Trial protocol rather than original article. |
| 56 | Cevasco 2008 | The outcome of interest was not physiological or behavioral. |
| 57 | Chang 2005 | It is not a randomized clinical trial. |
| 58 | Chorna 2014 | The outcome of interest was not physiological or behavioral. |
| 59 | Chou 2003 | It is not a randomized clinical trial. |
| 60 | Claire M 2018 | Cohort study rather than original article. |
| 61 | Clemencic-Jones 2024 | A systematic review rather than original article. |
| 62 | Corrigan 2020 | Effect sizes are reported as figures, not numerical values. |
| 63 | Costa 2022 | A meta-analysis article rather than original article. |
| 64 | Crave 1995 | It is not a randomized clinical trial about music therapy. |
| 65 | Da Silva 2013 | There was no control group data. |
| 66 | Da?li 2022 | The research sample was not pre-term neonates, but their mothers. |
| 67 | Dagli 2021 | The research sample was not pre-term neonates, but their mothers. |
| 68 | Darcy 2008 | A descriptive study rather than randomized clinical trial. |
| 69 | Dearn 2014 | The outcomes and effect sizes reported were inconsistent with the present study. |
| 70 | Dereddy 2024 | The outcomes and effect sizes reported were inconsistent with the present study. |
| 71 | Desquiotz-Sunnen 2008 | It is not a randomized clinical trial. |
| 72 | Detmer 2020 | The outcome of interest was not physiological or behavioral. |
| 73 | Dewan 2024 | The research sample was not pre-term neonates, but their parents. |
| 74 | Discenza 2013 | It is not a randomized clinical trial, also, the outcome of interest was not physiological or behavioral. |
| 75 | Djordjevic 2007 | It is not a randomized clinical trial about music therapy. |
| 76 | Dorn 2014 | The outcome of interest was not physiological or behavioral. |
| 77 | Drazen 2023 | It is not a randomized clinical trial about music therapy. |
| 78 | D'Souza 2017 | Trial protocol rather than original article. |
| 79 | Edalati 2023 | It is not a randomized clinical trial, also, the outcome of interest was not physiological or behavioral. |
| 80 | Emery 2019 | The outcome of interest was not physiological or behavioral. |
| 81 | Engeseth 2020 | It is not a randomized clinical trial, also, the outcome of interest was not physiological or behavioral. |
| 82 | Epstein 2021 | The outcome of interest was not physiological or behavioral. |
| 83 | Erdei 2024 | An case crossover study rather than randomized clinical trial. |
| 84 | Fajolu 2023 | A systematic review rather than original article. |
| 85 | Farhat 2010 | Effect sizes are reported as figures, not numerical values. |
| 86 | Fellman 2017 | An editorial article rather than original article. |
| 87 | Field 2006 | It is not a randomized clinical trial, also, the outcome of interest was not physiological or behavioral. |
| 88 | Filippa 2017 | A systematic review rather than original article. |
| 89 | Filippa 2018 | The outcome of interest was social not physiological or behavioral. |
| 90 | Franco 2024 | An cross-sectional study rather than randomized clinical trial. |
| 91 | Gaden 2022 | The outcome of interest was not physiological or behavioral. |
| 92 | Gao 2021 | The outcome of interest was not physiological or behavioral. |
| 93 | Gerber 1985 | A systematic review rather than original article. |
| 94 | Ghetti 2019 | Trial protocol rather than original article. |
| 95 | Ghetti 2021 | A cohort study rather than randomized clinical trial. |
| 96 | Giordano 2021 | The outcome of interest was not physiological or behavioral. |
| 97 | Gortner 2015 | The outcome of interest was not physiological or behavioral. |
| 98 | Graven 2000 | A systematic review rather than original article. |
| 99 | Haffner 1990 | It is not a randomized clinical trial about music therapy. |
| 100 | Haffner 1993 | It is not a randomized clinical trial about music therapy. |
| 101 | Haffner 1996 | It is not a randomized clinical trial about music therapy. |
| 102 | Hartling 2009 | A systematic review rather than original article. |
| 103 | Hasegawa 2020 | It is not a randomized clinical trial. |
| 104 | Haslbeck 2018 | It is not a randomized clinical trial. |
| 105 | He 2021 | A meta-analysis article rather than original article. |
| 106 | Heijden 2016 | A systematic review rather than original article. |
| 107 | Hodges 2010 | Research letter rather than original article. |
| 108 | Hoogen 2017 | A systematic review rather than original article. |
| 109 | Howard 2020 | The outcomes and effect sizes reported were inconsistent with the present study. |
| 110 | Hussey 2009 | The outcome of interest was not physiological or behavioral. |
| 111 | Jansson 2011 | Effect sizes are reported as figures, not numerical values. |
| 112 | Johnston 2007 | Effect sizes are reported as figures, not numerical values. |
| 113 | Juarez 2019 | Trial protocol rather than original article. |
| 114 | Kanagasabai 2013 | The outcome of interest was not physiological or behavioral. |
| 115 | Kaplan 2010 | The research sample was not pre-term neonates. |
| 116 | Karimi 2020 | The research sample was not pre-term neonates, but their mothers. |
| 117 | Katz 1971 | It is not a randomized clinical trial. |
| 118 | Kehl 2020 | Effect sizes are reported as figures, not numerical values. |
| 119 | Keidar 2014 | The outcome of interest was not physiological or behavioral. |
| 120 | Keith 2009 | The outcome of interest was not physiological or behavioral. |
| 121 | Kemper 2004 | A qualitative study rather than randomized clinical trial. |
| 122 | Kemper 2008 | The outcome of interest was not physiological or behavioral. |
| 123 | Khosla 2002 | It is not a randomized clinical trial about music therapy. |
| 124 | Khurana 2015 | There was no music therapy arm. |
| 125 | Kim 2021 | An observational study rather than randomized clinical trial. |
| 126 | Kjeldsen 2023 | The outcome of interest was not physiological or behavioral. |
| 127 | Kledmanee 2019 | A cohort study rather than randomized clinical trial. |
| 128 | Kobus 2021 | Effect sizes are reported as figures, not numerical values. |
| 129 | Koelsch 2014 | It is not a randomized clinical trial about music therapy. |
| 130 | Kostilainen 2021 | Effect sizes are reported as figures, not numerical values. |
| 131 | Kraft 2021 | Effect sizes are reported as figures, not numerical values. |
| 132 | Kramari? 2017 | It is not a randomized clinical trial. |
| 133 | Kritzinger 2014 | It is not a randomized clinical trial about music therapy. |
| 134 | Kupelian 2010 | The outcome of interest was not physiological or behavioral. |
| 135 | Laaksonen 2003 | There was no music therapy arm. |
| 136 | Lafferty 2023 | It is a randomized clinical trial, but the outcome of interest was not physiological or behavioral. |
| 137 | Lausanne 2019 | A book rather than original article. |
| 138 | Lejeune 2016 | Effect sizes are reported as figures, not numerical values. |
| 139 | Lejeune 2019 | The outcome of interest was not physiological or behavioral. |
| 140 | Liao 2018 | A systematic review rather than original article. |
| 141 | Liao 2021 | Sleep was monitored, but sleep darnation could not be calculated according to the data in the article. |
| 142 | Liwang 2018 | A systematic review rather than original article. |
| 143 | Loewy 2013 | Effect sizes are reported as figures, not numerical values. |
| 144 | Longcope 1990 | It is not a randomized clinical trial about music therapy. |
| 145 | Loscalzo 2023 | A pre-post study rather than randomized clinical trial. |
| 146 | Loukovaara 1995 | It is not a randomized clinical trial about music therapy. |
| 147 | Lubetzky 2010 | The outcome of interest was not physiological or behavioral. |
| 148 | Maggio 2006 | It is not a randomized clinical trial about music therapy. |
| 149 | Maguire 2014 | It is not a randomized clinical trial about music therapy. |
| 150 | Mahdieh 2021 | The outcome of interest was not physiological or behavioral. |
| 151 | Maitre 2020 | Randomization was not done. |
| 152 | Malinova 2004 | It is not a randomized clinical trial about music therapy. |
| 153 | Malloch 2012 | Effect sizes are reported as figures, not numerical values. |
| 154 | Martínez Ibarguen 1992 | It is not a randomized clinical trial about music therapy. |
| 155 | Martínez-Shaw 2023 | A systematic review rather than original article. |
| 156 | Maulik 2009 | A systematic review rather than original article. |
| 157 | Meder 2021 | Cohort study rather than original article. |
| 158 | Melo 2017 | It is not a randomized clinical trial. |
| 159 | Menke 2021 | The outcomes and effect sizes reported were inconsistent with the present study. |
| 160 | Mohan 2021 | A systematic review rather than original article. |
| 161 | Moher 2010 | A meta-analysis article rather than original article. |
| 162 | Moran 2015 | Effect sizes are reported as figures, not numerical values. |
| 163 | Nakhai-Pour 2007 | It is not a randomized clinical trial about music therapy. |
| 164 | Nasimi 2020 | There was no music therapy arm. |
| 165 | Neel 2019 | Trial protocol rather than original article. |
| 166 | O'Toole 2017 | A qualitative study rather than original article. |
| 167 | Ou 2024 | A meta-analysis article rather than original article. |
| 168 | Palazzi 2021 | The research sample was not pre-term neonates, but their mothers. |
| 169 | Pasquali 1995 | It is not a randomized clinical trial about music therapy. |
| 170 | Pavel 2019 | A congress abstract rather than original article, which had no usable information. |
| 171 | Peiris 1993 | It is not a randomized clinical trial about music therapy. |
| 172 | Peng 2018 | A congress abstract rather than original article, which had no usable information. |
| 173 | PLYMATE 1988 | It is not a randomized clinical trial about music therapy. |
| 174 | Provasi 2021 | Effect sizes are reported as figures, not numerical values. |
| 175 | Qiu 2017 | Effect sizes are reported as figures, not numerical values. |
| 176 | Ranger 2018 | Report median instead of mean. |
| 177 | Rehn 2002 | An animal study rather than randomized controlled trial. |
| 178 | Ren 2021 | Effect sizes are reported as figures, not numerical values. |
| 179 | Ribeiro 2018 | The outcomes and effect sizes reported were inconsistent with the present study. |
| 180 | Richard 2022 | The outcomes and effect sizes reported were inconsistent with the present study. |
| 181 | Roa 2018 | The research sample was not pre-term neonates, but their parents. |
| 182 | Sanchez 2018 | An editorial article rather than original article. |
| 183 | Sanchez 2019 | An editorial article rather than original article. |
| 184 | Schneider 2009 | A cross-sectional study rather than randomized controlled trial. |
| 185 | Shoemark 2015 | It is not a randomized clinical trial. |
| 186 | Shokri 2023 | The outcome of interest was not physiological or behavioral. |
| 187 | Span 2021 | Effect sizes are reported as figures, not numerical values. |
| 188 | Standley 1995 | It is not a randomized clinical trial. |
| 189 | Standley 1998 | It is not a randomized clinical trial. |
| 190 | Standley 2003 | The outcome of interest was not physiological or behavioral. |
| 191 | Standley 2010 | The outcome of interest was not physiological or behavioral. |
| 192 | Standley 2011 | It is not a randomized clinical trial. |
| 193 | Stegemann 2019 | An overview rather than randomized clinical trial. |
| 194 | Stouffer 2023 | A descriptive study rather than randomized clinical trial. |
| 195 | Sun 2020 | Trial protocol rather than original article. |
| 196 | Suwanrath 2018 | It is not a randomized clinical trial about music therapy. |
| 197 | Tan 2017 | It is not a randomized clinical trial about music therapy. |
| 198 | Tang 2007 | It is not a randomized clinical trial about music therapy. |
| 199 | Tengstrand 2002 | It is not a randomized clinical trial about music therapy. |
| 200 | Teunis 2017 | A systematic review rather than original article. |
| 201 | Thatrimontrichai 2009 | It is not a randomized clinical trial about music therapy. |
| 202 | Tsai 2004 | It is not a randomized clinical trial about music therapy. |
| 203 | Vaivre-Douret 2009 | There was no music therapy arm. |
| 204 | van der Heijden 2016 | A systematic review rather than original article. |
| 205 | van Dokkum 2020 | It is not a randomized clinical trial. |
| 206 | Vari?o?lu 2020 | The outcome of interest was not physiological or behavioral. |
| 207 | Varisco 2022 | It is not a randomized clinical trial. |
| 208 | Ventura 2012 | The research sample was not pre-term neonates, but pregnant women. |
| 209 | Vignozzi 2012 | It is not a randomized clinical trial about music therapy. |
| 210 | Virtala 2018 | A review article rather than original article. |
| 211 | Vitale 2021 | A review article rather than original article. |
| 212 | Walworth 2009 | Effect sizes are reported as figures, not numerical values. |
| 213 | Walworth 2012 | The outcome of interest was not physiological or behavioral. |
| 214 | Wang 2020 | It is not a randomized clinical trial about music therapy. |
| 215 | Whipple 2000 | The outcome of interest was not physiological or behavioral. |
| 216 | Wirth 2016 | Effect sizes are reported as figures, not numerical values. |
| 217 | Yakobson 2020 | Trial protocol rather than original article. |
| 218 | Yakobson 2021 | Effect sizes are reported as figures, not numerical values. |
| 219 | Yinger 2017 | A book without effect size rather than original article. |
| 220 | Yki-J?rvinen 1995 | It is not a randomized clinical trial about music therapy. |
| 221 | Yue 2021 | A meta-analysis article rather than original article. |
| 222 | Yurkovich 2018 | It is not a randomized clinical trial. |
| 223 | Zhang 2013 | It is not a randomized clinical trial about music therapy. |
| 224 | Zhi 2024 | a bibliometric and visualized study rather than randomized controlled trial. |
