# Supplementary material for: Effect of music therapy on behavioral and physiological neonatal outcomes: A systematic review and dose-response meta-analysis
Source: PLoS One. 2025 Jan 8;20(1):e0316674. doi: 10.1371/journal.pone.0316674 (PMC11709260; doi:10.1371/journal.pone.0316674)
Supplement: Attachment — Submitted filename: PONE-D-23-42926_R2 RV.pdf [file pone.0316674.s007.pdf]

PLOS ONE

Effect of music therapy on behavioral and physiological neonatal outcomes: A systematic review and Dose-Response Meta-analysis

--Manuscript Draft--

**Manuscript Number:**	PONE-D-23-42926R2
**Article Type:**	Research Article
**Full Title:**	Effect of music therapy on behavioral and physiological neonatal outcomes: A systematic review and Dose-Response Meta-analysis
**Short Title:**	Effect of music therapy on neonatal outcomes
**Corresponding Author:**	Marjan Shahbazi<br>Iran University of Medical Sciences: Tehran University of Medical Sciences<br>IRAN, ISLAMIC REPUBLIC OF
**Keywords:**	Music therapy, preterm infant, neonatal, Randomized Controlled Trials, meta-analysis, systematic review.
**Abstract:**	Background: Previous studies have documented the effectiveness of music therapy in treating adverse neonatal outcomes in premature neonates. However, how long listening to music can improve neonatal outcomes is a question addressed in this review.<br>Methods: For doing this dose-response meta-analysis, we searched PubMed, Scopus, Web of Sciences, and Cochrane Library databases. The inclusion criteria were randomized clinical trials that investigated the effects of music therapy on improving adverse neonatal outcomes. Preterm babies were those who were born between 27 and 37 weeks of pregnancy (Fetuses respond to auditory stimuli from the 27th week of pregnancy). Also, the initial search was done on Juan 28, 2024 and the start and end time of the search was not limited either. Finally, we used a two-stage random effect model by “drmeta” package in Stata software to run this dose-response meta-analysis.<br>Results: In total, 37 articles (2082 participants) were identified for inclusion in our meta-analysis. According to pooled analysis with each minute increase in music therapy, the means of respiratory rate, pain score, SBP, DBP, behavioral score, and body temperature decrease by 35.3 beats per minutes, 15.3 VAS, 30.7 mmHg, 8.9 mmHg, 2.7, and 0.27 ºC. On the other hand, with each minute increase in listening to the music, the mean of O2 saturation, heart rate and sleep duration increase 1.7%, 89.2 beats per minutes and 5.081 minutes per day, respectively.<br>Conclusion: Music therapy improves the neonatal outcomes of O2 saturation, heart rate, respiratory rate, sleep duration, body temperature and systolic and diastolic blood pressures. Therefore, the existence of a dose-response relationship can indicate a causal relationship between music therapy and the improvement of neonatal outcomes.
**Order of Authors:**	Fatemeh Shahbazi
	Marziyeh Fattahi-Darghlou
	Samad Moslehi
	Minoo Dabiri-Golchin
	Marjan Shahbazi
**Response to Reviewers:**	Dear editor,<br>we have corrected all the comments of reviewer number 1 and sent back to you the revised article in the same format as you said.
**Additional Information:**	
**Question**	**Response**
**Financial Disclosure**<br><br>Enter a financial disclosure statement that	The author(s) received no specific funding for this work.

Powered by Editorial Manager® and ProduXion Manager® from Aries Systems Corporation

Manuscript Click here to access/download;Manuscript;Manuscript.docx

Effect of music therapy on behavioral and physiological neonatal outcomes: A systematic review and Dose-Response Meta-analysis

Fatemeh Shahbazi[1, 2], Marzieh Fattahi-Darghlou[1], Samad Moslehi[3], Minoo Dabiri-Golchin[4], Marjan Shahbazi[5*]

[1] Department of Epidemiology, School of Public Health, Hamadan University of Medical Sciences, Hamadan, Iran.

[2] Occupational health and safety research center, Health Sciences & Technology Research Institute, Hamadan University of Medical Sciences, Hamadan, Iran.

[3] Department of Biostatistics, School of Public Health, Hamadan University of Medical Sciences, Hamadan, Iran.

[4] Department of Occupational Therapy, University of Manitoba Winnipeg, MB, Canada.

[5] Department of Occupational Therapy, School of Rehabilitation Sciences, Iran University of Medical Sciences, Tehran, Iran.

*Corresponding author: Marjan Shahbazi, Email: marjanshahbazi55@yahoo.com Tel: +988138380025

Abstract

Background: Previous studies have documented the effectiveness of music therapy in treating adverse neonatal outcomes in premature neonates. However, how long listening to music can improve neonatal outcomes is a question addressed in this review.

Methods: For doing this dose-response meta-analysis, we searched PubMed, Scopus, Web of Sciences, and Cochrane Library databases. The inclusion criteria were randomized clinical trials that investigated the effects of music therapy on improving adverse neonatal outcomes. Preterm babies were those who were born between 27 and 37 weeks of pregnancy (Fetuses respond to auditory stimuli from the 27th week of pregnancy). Also, the initial search was done on Juan 28, 2024 and the start and end time of the search was not limited either. Finally, we used a two-stage random effect model by "drmeta" package in Stata software to run this dose-response meta-analysis.

Results: In total, 37 articles (2082 participants) were identified for inclusion in our meta-analysis. According to pooled analysis with each minute increase in music therapy, the means of respiratory rate, pain score, SBP, DBP, behavioral score, and body temperature decrease by 35.3 beats per minutes, 15.3 VAS, 30.7 mmHg, 8.9 mmHg, 2.7, and 0.27 ºC. On the other hand, with each minute increase in listening to the music, the mean of O2 saturation, heart rate and sleep duration increase 1.7%, 89.2 beats per minutes and 5.081 minutes per day, respectively.

Conclusion: Music therapy improves the neonatal outcomes of O2 saturation, heart rate, respiratory rate, sleep duration, body temperature and systolic and diastolic blood pressures. Therefore, the existence of a dose-response relationship can indicate a causal relationship between music therapy and the improvement of neonatal outcomes.

Keywords: Music therapy, preterm infant, neonatal, Randomized Controlled Trials, meta-analysis, systematic review.

Introduction

Music therapy is the clinical and evidence-based use of music interventions with organized melody, rhythm, harmony, and timbre to improve health outcomes (1, 2). Music is often considered an auxiliary medical treatment due to its involvement in physiological, psychological, and social functions (3-5). It can also influence the nervous system's cortical, subcortical, and vegetative areas, strengthen psycho-physiological defenses, and improve health (6). The benefits of Music therapy for treating adverse neonatal outcomes have been investigated in previous research (7, 8). Music therapy has shown the ability to improve and stabilize newborns' vital signs and physiological responses (4, 9). This intervention can improve premature babies' sleep duration, body temperature, oxygen arterial saturation, and behavioral scores. It can also reduce pain and anxiety levels (10).

In recent years, clinicians and neonatologists have been using music therapy for the reasons of low cost, being non-invasiveness, and non-pharmacological for premature infants hospitalized in the NICU (10, 11). Based on the findings of previous single studies, music therapy has played an effective role in improving various conditions of premature neonates such as heart rate, breathing rate, oxygen saturation, and the functioning of the parasympathetic nervous system (12). Even in some studies, it was found that combining it with some care methods such as kangaroo care has relieved the pain of infants; however, the results are inconsistent (13, 14). A meta-analysis in 2012 showed the effectiveness of music in infants admitted to the ICU, but this study had two serious limitations: first, it included only articles that were published in English, and second, the included studies were mostly observational not randomized clinical trials (15). Recently, Yue et al published a systematic review and meta-analysis and concluded that music therapy can significantly effect on improving the heart and respiratory rates and stress level of premature infants (10). The recent meta-analysis only examined the outcomes of heart rate, respiratory rate, oxygen saturation, behavioral state, and stress level, while the present meta-analysis investigate nine physiological and behavioral neonatal outcomes. On the other hand, after the latest meta-analysis, new randomized clinical trials have been published on music therapy and neonatal conditions; therefore, an updated systematic review and meta-analysis is needed. In addition, this synthesis focused on dose–response relationship between massage therapy and neonatal conditions for the first time. Consequently, this systematic review examines the dose-response relationship between music therapy and neonatal outcomes in preterm infants.

Method

Eligibility criteria (PICOS)

Population: The study population comprised preterm infants who were born between 27 and 37 weeks of gestation (fetuses respond to auditory stimuli from the 27th week of pregnancy). Intervention: The intervention group included neonates who were hospitalized in the neonatal intensive care unit (NICU) and received music therapy.

Control: The control group consisted of neonates who received usual care in NICU.

Outcome: The primary outcome was neonatal status. Neonatal status included heart rate (beats per minute), systolic and diastolic blood pressures (mmHg), respiratory rate (breaths per minute), pain score (visual analog scale (VAS)), behavioral score, sleep duration (hours per day), oxygen arterial saturation (%), and body temperature (ºC). Also, music therapy is an auxiliary and palliative treatment, and according to the literature review, there were no secondary outcomes and side effects to reporting.

Studies: Randomized controlled trials were included in the analysis regardless of language and their publication status. These studies specifically investigated the effectiveness of music therapy on neonatal status. To be eligible for inclusion, studies were required to present the mean difference (MD) along with the corresponding 95% confidence interval (CI), or provide sufficient information to calculate the effect sizes manually.

Confounding control

We included randomized controlled trials. Randomization in clinical trials is a method of adjusting confounder variables in experimental studies. In other words, randomization makes the studied groups to be balanced in terms of known and unknown confounders. For this reason, we only examined the experimental studies that investigated the effects of music therapy with a randomized design.

Information source and search strategy

We searched four electronic databases for eligible studies: PubMed, Scopus, Web of Science, and Cochran Library. The search was conducted on Juan 28, 2024, and its start date was unlimited. We searched varying combinations of keywords and Mesh terms (((((((((premature infant) OR (infant,

premature)) OR (prematurity, neonatal)) OR (LBW)) OR (low birth weight)) OR (premature infant*)) OR (preterm infant*)) OR (prematurity)) AND (((((((music therapy) OR (auditory stimulation)) OR (acoustic stimulation)) OR (music)) OR (song*)) OR (singing*)) OR (therapy, music))) AND ((((randomized controlled trial)) OR (RCT)) OR (placebo)) OR (random)). We also manually screened the reference lists of all relevant articles to find additional articles that might have been missed in the systematic search. Duplicate reports of the same study were deleted.

Selection and data collection process

We used EndNote software (version X8) for Study screening and selection. Two investigators (MF and MSH) independently screened all non-duplicate titles and abstracts, evaluated the full texts for eligibility, and performed data extraction using a predesigned data form in Stata software. Any discrepancies between authors were resolved by discussion or consensus with an expert (FSH).

Data items

The data extracted from all included studies were author's first name, publication year, outcome, country, number of participants, mean difference, standard deviation, type of trial (parallel RCTs and crossover RCTs), type of music (live or recorded music), and duration of music playback during the trial (per minutes in day).

Determining the dose of music

To determine the dose of music for each study, we first decided how many days the preterm infants received music therapy in each trial. In the next step, we determined how many minutes they listened to music each day. In the last stage, the final dose was determined by multiplying the number of intervention days by the number of minutes of listening to music.

Quality assessment of studies

We used the Cochrane criteria for RCT studies to assess the methodological quality of the included studies. We evaluated the quality of all relevant studies based on random sequence generation (selection bias due to inadequate generation of a randomized sequence), allocation concealment (selection bias due to insufficient concealment of allocations before assignment), blinding of participants and personnel (performance bias due to knowledge of the allocated interventions by participants and personnel during the study), blinding of outcome assessment (detection bias due to knowledge of the allocated interventions by outcome assessor), incomplete outcome data

(attrition bias due to amount, nature or handling of incomplete outcome data), and selective reporting (reporting bias due to selective outcome reporting). Then, the included RCTs were classified as low risk of bias if all mentioned criteria were met, unclear risk bias if one item was not met, and high risk of bias if more than one item was not fulfilled.

Statistical analysis

We performed a random-effects dose-response meta-analysis to assess the relationship between changes in the duration of music and the changes in neonatal outcomes using the two-stage mixed-effect meta-analysis model. This method uses the "drmeta" command in Stata software version 17 (Stata Corp LLC, Texas, US). Greenland and Orsini described this method in 2006 (16). This model used linear, quadratic, and cubic spline dose-response meta-analysis to evaluate the relationship between music and neonate status. We must note that we used restricted cubic splines of music therapy with three knots at fixed percentiles (25%, 50%, and 78%), having no a priori assumptions regarding the shape of the association. So, different models were fitted to the data, and the best model was chosen based on the Akaike information Criteria (AIC) and Bayesian Information Criteria (BIC).

We extracted the mean and standard deviation of neonatal outcomes in each arm of RCT that was measured based on the minutes. Then, we defined the mean difference in neonate outcomes after the intervention as the difference at the end minus the corresponding baseline value in the active and control arms of the trial. For this dose-response meta-analysis, the total duration of the music therapy per day was set as the midpoint in each category. We used the Egger test (17) and funnel plot (18) to examine the possibility of publication bias . The software Stata version 17, and Review Manager 5.4 were used for data analysis.

Results

Description of studies

The PRISMA (Preferred Reporting Items for Systematic Review and Meta-Analysis) literature search flowchart is presented in Figure 1. We retrieved 254 titles and abstracts, and after their screening, only 56 articles met our inclusion criteria. Finally, after we read their full text, only 37 randomized clinical trials remained in the final analysis. Reasons for the exclusion of the articles were duplicate reports on the same population (n=4), no trial with music intervention (n=7), and previous meta-analyses, systematic reviews, and narrative reviews about the effect of music

therapy on neonatal outcomes (n=8). Table 1 presents selected characteristics of the 37 trials included in the analysis. We investigated 933 neonates in the intervention group and 1149 cases in the control group. Among the included trials, there were 24 studies in Asia, 4 studies in America, 7 studies in Europe and 2 studies in Australia. All trials reported adjusted effect sizes and were conducted in both sexes. The neonate's outcomes distribution was as follows: heart rate (27 trials), respiratory rate (19 trials), O2 saturation (29 trials), pain score (14 trials), body temperature (2 trials), behavioral score (7 trials), systolic and diastolic blood pressures (2 trial), and sleep duration (2 trial). The characteristics of the included studies are given in Table 1.

Dose-response meta-analysis

With the increase of each minute of music therapy, the average of respiratory rate, pain score, SBP, DBP, behavioral score, and body temperature decrease by -0.353 (95% CI: -0.409 to -0.297), -0.153 (95% CI: -0.169 to -0.136), -0.307 (95% CI: -0.497 to -0.118), -0.089 (95% CI: -0.287 to 0.108), -0.027 (95% CI: -0.031 to -0.012), and -0.002 (95% CI: -0.004 to -0.0007). On the other hand, for each minute increase in music therapy, the mean difference (mean in the group receiving music minus the group not receiving it) of respiratory rate, pain score, SBP, DBP, behavioral score, and body temperature decrease by 35.3 beats per minutes, 15.3 VAS, 30.7 mmHg, 8.9 mmHg, 2.7, and 0.27 ºC. Also, with the increase of every minute of music therapy, the mean difference of O2 saturation, heart rate and sleep duration increase by 0.017 (95% CI: 0.013 to 0.021),0.892 (95% CI: 0.868 to 0.917), and 5.081 (95% CI: 4.910 to 5.253). On the other hand, with each minute increase in listening to the music, the mean difference (mean in the group receiving music minus the group not receiving it) of O2 saturation, heart rate and sleep duration increase 1.7%, 89.2 beats per minutes and 5.081 minutes per day, respectively (Figure 2).

Methodological quality and risk of bias for included trials

As shown in the risk of bias assessment tool in Figure 3, the methodological quality of the included studies was acceptable. All studies included in this meta-analysis were classified as low-risk categories regarding incomplete outcome data, selective reporting, and other biases. As for the random sequence generation of included studies, more than three-quarters of included trials mentioned generating a randomized sequence. Allocation concealment was not mentioned in seven studies, but four detailed the use of computer-based randomization, two detailed the use of drawing

lots, and three detailed the use of a random number table to generate the random sequence. Because of the nature of music therapy intervention, less than half of the trials successfully conducted the double-blinding methods.

Publication bias and quality of the evidence

In our study, the Egger test result indicated no significant publication bias in included trials that investigated the effect of music therapy on neonatal outcomes ($p = 0.294$). There was no significant publication bias when we measured the Egger test for measured results. The significance values of the Egger test for included outcomes were as follows: behavioral score: 0.052, DBP: 0.317, heart rate: 0.083, O2 saturation: 0.925, pain score: 0.988, respiratory rate: 0.227, SBP: 0.317, sleep duration: 0.207, and body temperature: 0.534. Also, we assessed publication bias using the funnel plot. The symmetry distribution of studies around the null line in all funnel plots indicates the absence of publication bias (Figure 4).

Discussion

This dose-response meta-analysis revealed that increased music therapy per minute is significantly associated with improved O2 saturation, heart rate, respiratory rate, sleep duration, body temperature, and systolic and diastolic pressure in preterm babies. This evidence shows a dose-response relationship between music therapy and neonatal outcomes. When a dose-response relationship is present, it adds plausibility to a causal relationship between exposure and outcome (19).

This meta-analysis identified its clinical value in improving preterm infant's O2 saturation, heart rate, respiratory rate, blood pressure, sleep duration, and body temperature. In justification of these findings, it can be said as follows: Studies in music and neuroscience demonstrate that music promotes neurobiological processes and modulates synaptic plasticity, neuronal learning, and readjustment in the human brain (20). Music activates limbic and paralimbic structures (21). Particularly in newborns, music promotes neuronal activation, and this activation may enhance psychological and physiological health. With premature birth, the intrauterine sheltering environment perfect for fetal growth and maturation is abandoned too early. Besides other stressful experiences, such as the separation from the mother and painful procedures, preterm infants must cope with the unusual sound environment of an intensive care unit (20). Proper NICU levels are

often 50–80 dB, sometimes reaching a peak of 120 dB (22). However, the American Academy of Pediatrics recommended that sound levels not exceed 45 dB for neonatal intensive care units (23). Preterm infants are susceptible to noise because their auditory system and brain development are in a critical, vulnerable, and fast period of growth. Stressful noise can further initiate stress responses in preterm infants. The sympathetic autonomic nervous system and the hypothalamic-pituitary-adrenal axis of the endocrine system are activated, known as the "fight or flight" reaction. This response may use energy reserves crucial for preterm infants' brain development (24). Thus, the overwhelming auditory neonatal intensive care environment is assumed to interfere with the short- and long-term neurobehavioral growth in preterm infants (25). To summarize, the reason for the effect of music on physiological responses is explained by the fact that music, by affecting the nervous system (limbic and autonomic system), produces a relaxation response, which is characterized by regulation of breathing rate and heart rate and increased oxygen saturation.

Infants born preterm engage in lighter and more active sleep than infants born at term. Sleep patterns may be significant for preterm infants, who often exhibit signs of bio behavioral disorganization during daytime activities (26). A study by Schwichtenberg and colleagues found that preterm infants appeared to benefit from more "breaks" (i.e., naps) during the day to reorganize and maintain their engagement in the social environment. Infants who are more rested during the day are more regulated and able to benefit from positive parental behaviors over time. In a way, it strengthens the attachment of the baby and the mother (27). Since the effect on the brain mediates music therapy by repeatedly inducing alpha brain waves during the waking period, it increases sleep duration in preterm infants (28). In this way, the amount of infant-mother attachment rises (26). Also, suppose music therapy in neonatal care is a family-integrated approach in which each family is encouraged to find their innate way of relating to their newborn through music. In that case, it may empower parents by enhancing their well-being, self-confidence, and quality of interactions with their newborn (20).

In our meta-analysis, music therapy has statistically significant impact on pain reduction and the behavioral state of premature infants. The reason for the significant effect of music on reduction of pain and behavioral scores may be because the studies that investigated the impact of music therapy on pain and behavior reduction were conducted during invasive procedures such as blood

sampling, which is effective in reducing these two variables in preterm infants by improving physiological responses (29, 30).

Another interesting finding of this synthesis was that no trial had been conducted on the effectiveness of music therapy in improving the physiological and behavioural conditions of preterm infants in the African region. This is while according to the latest global burden of diseases study in 2019, the highest incidence of neonatal preterm birth was in sub-Saharan Africa (31). Therefore, it is recommended that randomized clinical trials investigate the effectiveness of music therapy on neonatal outcomes in the African region.

This meta-analysis had several limitations and potential flaws. First, some studies seemed potentially eligible for inclusion in our meta-analysis, but we needed help accessing their full text. This problem can increase the possibility of selection bias. Second, in the randomized clinical trials included in our meta-analysis, some participants discontinued the study due to the length of follow-up. This can lead to a selection bias in our results. Third, the RCTs' varying music therapy modalities (from live music to recorded music) may affect the pooled results. The most important strength of this study is that it allows us to know how much listening to music improved neonatal outcomes in neonates. In addition, the broad search strategy used in this study increased the sensitivity of the search to include as many relevant articles as possible. Finally, an important point that should be mentioned is that our study examined more neonatal outcomes than Wei Yue's study in 2020 (10). On the other hand, Wei's study was a simple meta-analysis, but ours was a dose-response meta-analysis.

Conclusion

In conclusion, music therapy improves neonatal outcomes of O2 saturation, heart rate, respiratory rate, sleep duration, body temperature, and systolic and diastolic pressure. The existence of a dose-response relationship between music therapy and the mentioned neonate outcomes can strengthen the scientific background for therapeutic intervention in the newborn intensive care unit (NICU) for the treatment of adverse neonatal outcomes. There is no association between music therapies and pain score and behavioral score.

References

1. Yinger OS. Music therapy: Research and evidence-based practice: Elsevier Health Sciences; 2017.

2. Standley JM. Music therapy for the neonate. Newborn and Infant Nursing Reviews. 2001;1(4):211-6.

3. Teckenberg-Jansson P, Huotilainen M, Pölkki T, Lipsanen J, Järvenpää A-L. Rapid effects of neonatal music therapy combined with kangaroo care on prematurely-born infants. Nordic Journal of Music Therapy. 2011;20(1):22-42.

4. Loewy J, Stewart K, Dassler A-M, Telsey A, Homel P. The effects of music therapy on vital signs, feeding, and sleep in premature infants. Pediatrics. 2013;131(5):902-18.

5. Bieleninik Ł, Ettenberger M, Epstein S, Elefant C, Arnon S. Potential psychological and biological mechanisms underlying the effectiveness of neonatal music therapy during kangaroo mother care for preterm infants and their parents. International Journal of Environmental Research and Public Health. 2021;18(16):8557.

6. He H, Huang J, Zhao X, Li Z. The effect of prenatal music therapy on fetal and neonatal status: A systematic review and meta-analysis. Complementary Therapies in Medicine. 2021;60:102756.

7. Gaden TS, Ghetti C, Kvestad I, Bieleninik Ł, Stordal AS, Assmus J, et al. Short-term music therapy for families with preterm infants: a randomized trial. Pediatrics. 2022;149(2).

8. Dağli E, Çelik N. The effect of oxytocin massage and music on breast milk production and anxiety level of the mothers of premature infants who are in the neonatal intensive care unit: A self-controlled trial. Health care for women international. 2022;43(5):465-78.

9. Palazzi A, Meschini R, Piccinini CA. NICU music therapy effects on maternal mental health and preterm infant's emotional arousal. Infant Mental Health Journal. 2021;42(5):672-89.

10. Yue W, Han X, Luo J, Zeng Z, Yang M. Effect of music therapy on preterm infants in neonatal intensive care unit: Systematic review and meta-analysis of randomized controlled trials. Journal of Advanced Nursing. 2021;77(2):635-52.

11. Ou Y, Chen L, Zhu X, Zhang T, Zhou Y, Zou L, et al. The effect of music on pain management in preterm infants during daily painful procedures: a systematic review and meta-analysis. Frontiers in Pediatrics. 2024;12:1351401.

12. Sanfilippo K. Book Review: Music therapy in neonatal intensive care: Influences of culture (Shoemark & Ettenberger, Eds.). Approaches: An Interdisciplinary Journal of Music Therapy. 2021.

13. Span LC, van Dokkum NH, Ravensbergen A-G, Bos AF, Jaschke AC. Combining kangaroo care and live-performed music therapy: Effects on physiological stability and neurological functioning in extremely and very preterm infants. International Journal of Environmental Research and Public Health. 2021;18(12):6580.

14. Vahdati M, Mohammadizadeh M, Talakoub S. Effect of Kangaroo care combined with music on the mother–premature neonate attachment: A randomized controlled trial. Iranian journal of nursing and midwifery research. 2017;22(5):403-7.

15. Standley J. Music therapy research in the NICU: an updated meta-analysis. Neonatal Network. 2012;31(5).

16. Orsini N, Bellocco R, Greenland S. Generalized least squares for trend estimation of summarized dose–response data. The stata journal. 2006;6(1):40-57.

17. Egger M, Smith GD, Schneider M, Minder C. Bias in meta-analysis detected by a simple, graphical test. bmj. 1997;315(7109):629-34.

18. Sterne JA, Harbord RM. Funnel plots in meta-analysis. The stata journal. 2004;4(2):127-41.

19. Leon G. Epidemiology. 5th edition ed: Philadelphia: Saunders.; 2014.

20. Haslbeck FB, Bassler D. Music from the very beginning—a neuroscience-based framework for music as therapy for preterm infants and their parents. Frontiers in behavioral neuroscience. 2018;12:112.

21. Koelsch S. Brain correlates of music-evoked emotions. Nature reviews neuroscience. 2014;15(3):170-80.

22. Darcy AE, Hancock LE, Ware EJ. A descriptive study of noise in the neonatal intensive care unit: ambient levels and perceptions of contributing factors. Advances in Neonatal Care. 2008;8(5):S16-S26.

23. Etzel RA, Balk S. Noise: a hazard for the fetus and newborn. Pediatrics. 1997;100(4):724-7.

24. Lammertink F, Vinkers CH, Tataranno ML, Benders MJ. Premature birth and developmental programming: mechanisms of resilience and vulnerability. Frontiers in psychiatry. 2021;11:531571.
25. Wachman EM, Lahav A. The effects of noise on preterm infants in the NICU. Archives of Disease in Childhood-Fetal and Neonatal Edition. 2011;96(4):F305-F9.
26. Schwichtenberg A, Shah PE, Poehlmann J. Sleep and attachment in preterm infants. Infant mental health journal. 2013;34(1):37-46.
27. Schwichtenberg A, Anders TF, Vollbrecht M, Poehlmann J. Daytime sleep and parenting interactions in infants born preterm. Journal of Developmental & Behavioral Pediatrics. 2011;32(1):8-17.
28. Shah_farhat A, Kamarbandi S, Amiri R, Mohammadzadeh A. The effect of music on respiratory and heart rate of premature infants. Journal of Kermanshah University of Medical Sciences. 2008;12(3).
29. Alipour Z, Eskandari N, Hossaini SKE, Sangi S. Effects of music on physiological and behavioral responses of premature infants: a randomized controlled trial. Complementary therapies in clinical practice. 2013;19(3):128-32.
30. Ranger A, Helmert E, Bott T, Ostermann T, Als H, Bassler D, et al. Physiological and emotional effects of pentatonic live music played for preterm neonates and their mothers in the newborn intensive care unit: a randomized controlled trial. Complementary therapies in medicine. 2018;41:240-6.
31. Cao G, Liu J, Liu M. Global, regional, and national incidence and mortality of neonatal preterm birth, 1990-2019. JAMA pediatrics. 2022;176(8):787-96.
32. Alemdar DK, Özdemir FK. Effects of covering the eyes versus playing intrauterine sounds on premature infants' pain and physiological parameters during venipuncture. Journal of Pediatric Nursing. 2017;37:e30-e6.
33. Arnon S, Shapsa A, Forman L, Regev R, Bauer S, Litmanovitz I, et al. Live music is beneficial to preterm infants in the neonatal intensive care unit environment. Birth. 2006;33(2):131-6.
34. Barandouzi ZA, Keshavarz M, Montazeri A, Ashayeri H, Rajaei Z. Comparison of the analgesic effect of oral sucrose and/or music in preterm neonates: a double-blind randomized clinical trial. Complementary therapies in medicine. 2020;48:102271.

35. Caparros-Gonzalez RA, de la Torre-Luque A, Diaz-Piedra C, Vico FJ, Buela-Casal G. Listening to relaxing music improves physiological responses in premature infants: a randomized controlled trial. Advances in Neonatal Care. 2018;18(1):58-69.

36. Dehghani K, Movahed ZP, Dehghani H, Nasiriani K. A randomized controlled trial of kangaroo mother care versus conventional method on vital signs and arterial oxygen saturation rate in newborns who were hospitalized in neonatal intensive care unit. Journal of clinical neonatology. 2015;4(1):26-31.

37. Döra Ö, Büyük ET. RETRACTED: Effect of White Noise and Lullabies on Pain and Vital Signs in Invasive Interventions Applied to Premature Babies. Elsevier; 2021.

38. Howard C, Powell AS, Pavlidis E, Pavel A, Finn D, Allen A, et al. No effect of a musical intervention on stress response to venepuncture in a neonatal population. Acta Paediatrica. 2020;109(3):511-7.

39. Keith DR, Russell K, Weaver BS. The effects of music listening on inconsolable crying in premature infants. Journal of music therapy. 2009;46(3):191-203.

40. Kobus S, Diezel M, Dewan MV, Huening B, Dathe A-K, Felderhoff-Mueser U, et al. Music therapy is effective during sleep in preterm infants. International journal of environmental research and public health. 2021;18(16):8245.

41. Kurdahi Badr L, Demerjian T, Daaboul T, Abbas H, Hasan Zeineddine M, Charafeddine L. Preterm infants exhibited less pain during a heel stick when they were played the same music their mothers listened to during pregnancy. Acta Paediatrica. 2017;106(3):438-45.

42. Namjoo R, Mehdipour-Rabori R, Bagherian B, Nematollahi M. Comparing the effectiveness of mother's live lullaby and recorded lullaby on physiological responses and sleep of preterm infants: a clinical trial study. Journal of Complementary and Integrative Medicine. 2022;19(1):121-9.

43. Qolizadeh A, Myaneh ZT, Rashvand F. Investigating the effect of listening to the Holy Quran on the physiological responses of neonates admitted to neonatal intensive care units: A pilot study. Advances in Integrative Medicine. 2019;6(4):159-62.

44. Uematsu H, Sobue I. Effect of music (Brahms lullaby) and non-nutritive sucking on heel lance in preterm infants: A randomized controlled crossover trial. Paediatrics & child health. 2019;24(1):e33-e9.

45. Tandoi F, Francescato G, Pagani A, Buzzetti G, Negri E, Agosti M. “The Original Sound”: a new non-pharmacological approach to the postnatal stress management of preterm infants. The Journal of Maternal-Fetal & Neonatal Medicine. 2015;28(16):1934-8.
46. Amini E, Rafiei P, Zarei K, Gohari M, Hamidi M. Effect of lullaby and classical music on physiologic stability of hospitalized preterm infants: a randomized trial. Journal of neonatal-perinatal medicine. 2013;6(4):295-301.
47. Arnon S, Diamant C, Bauer S, Regev R, Sirota G, Litmanovitz I. Maternal singing during kangaroo care led to autonomic stability in preterm infants and reduced maternal anxiety. Acta paediatrica. 2014;103(10):1039-44.
48. Aydin D, Yildiz S. Effect of classical music on stress among preterm infants in a neonatal intensive care unit. HealthMED. 2012;6(9):3176-82.
49. Dur Ş, Çevik SG, Ustabaş Yıldız N. The effect of white noise and classical music on pain and physiologic parameters in preterm infants during retinopathy of prematurity examinations: a randomized controlled trial. Early Child Development and Care. 2023;193(1):60-71.
50. Garunkstiene R, Buinauskiene J, Uloziene I, Markuniene E. Controlled trial of live versus recorded lullabies in preterm infants. Nordic Journal of Music Therapy. 2014;23(1):71-88.
51. Jabraeili M, Sabet T, MustafaGharebaghi M, Jafarabadi MA, Arshadi M. The effect of recorded mum's lullaby and Brahm’s lullaby on oxygen saturation in preterm infants: A randomized double-blind clinical trial. Journal of caring sciences. 2016;5(1):85.
52. Lubetzky R, Mimouni FB, Dollberg S, Reifen R, Ashbel G, Mandel D. Effect of music by Mozart on energy expenditure in growing preterm infants. Pediatrics. 2010;125(1):e24-e8.
53. Olischar M, Shoemark H, Holton T, Weninger M, Hunt RW. The influence of music on aEEG activity in neurologically healthy newborns≥ 32 weeks’ gestational age. Acta Paediatrica. 2011;100(5):670-5.
54. Schlez A, Litmanovitz I, Bauer S, Dolfin T, Regev R, Arnon S. Combining kangaroo care and live harp music therapy in the neonatal intensive care unit setting. Néonatologie, interventions infirmières afin de gérer les émotions des parents. 2011:46.
55. Standley JM. The effect of music-reinforced nonnutritive sucking on feeding rate of premature infants. Journal of pediatric nursing. 2003;18(3):169-73.

56. Standley JM, Cassidy J, Grant R, Cevasco A, Szuch C, Nguyen J, et al. The effect of music reinforcement for non-nutritive sucking on nipple feeding of premature infants. Pediatric nursing. 2010;36(3):138.
57. Taheri L, Jahromi MK, Abbasi M, Hojat M. Effect of recorded male lullaby on physiologic response of neonates in NICU. Applied Nursing Research. 2017;33:127-30.
58. Tang L, Wang H, Liu Q, Wang F, Wang M, Sun J, et al. Effect of music intervention on pain responses in premature infants undergoing placement procedures of peripherally inserted central venous catheter: A randomized controlled trial. European Journal of Integrative Medicine. 2018;19:105-9.
59. Whipple J. The effect of music-reinforced nonnutritive sucking on state of preterm, low birthweight infants experiencing heelstick. Journal of Music Therapy. 2008;45(3):227-72.
60. Lai H-L, Chen C-J, Peng T-C, Chang F-M, Hsieh M-L, Huang H-Y, et al. Randomized controlled trial of music during kangaroo care on maternal state anxiety and preterm infants' responses. International journal of nursing studies. 2006;43(2):139-46.
61. Midilli TS, Ergin E. The effect of white noise and Brahms' lullaby on pain in infants during intravenous blood draw: a randomized controlled study. vital signs. 2023;6:11.
62. Mirzaee K, Jahanpour F, Gashmard R, Akaberian S. Comparison of the Effect of Music Therapy and Swaddling on Pain Intensity Caused by Blood Sampling in Premature Infants Admitted to the Neonatal Intensive Care Unit: A Randomized Trial. Iranian South Medical Journal. 2023;25(5):454-65.
63. Shah SR, Kadage S, Sinn J. Trial of music, sucrose, and combination therapy for pain relief during heel prick procedures in neonates. The Journal of pediatrics. 2017;190:153-8. e2.
64. Shukla VV, Bansal S, Nimbalkar A, Chapla A, Phatak A, Patel D, et al. Pain control interventions in preterm neonates: A randomized controlled trial. Indian pediatrics. 2018;55:292-6.
65. Yarahmadi S, Pouralizadeh M, Atrkarroushan Z, Shahroudi P. The effect of the simulated intrauterine sound on behavioral and physiological indices of pain during capillary blood sampling for screening preterm infants: a randomized clinical trial study. BMC pediatrics. 2024;24(1):110.

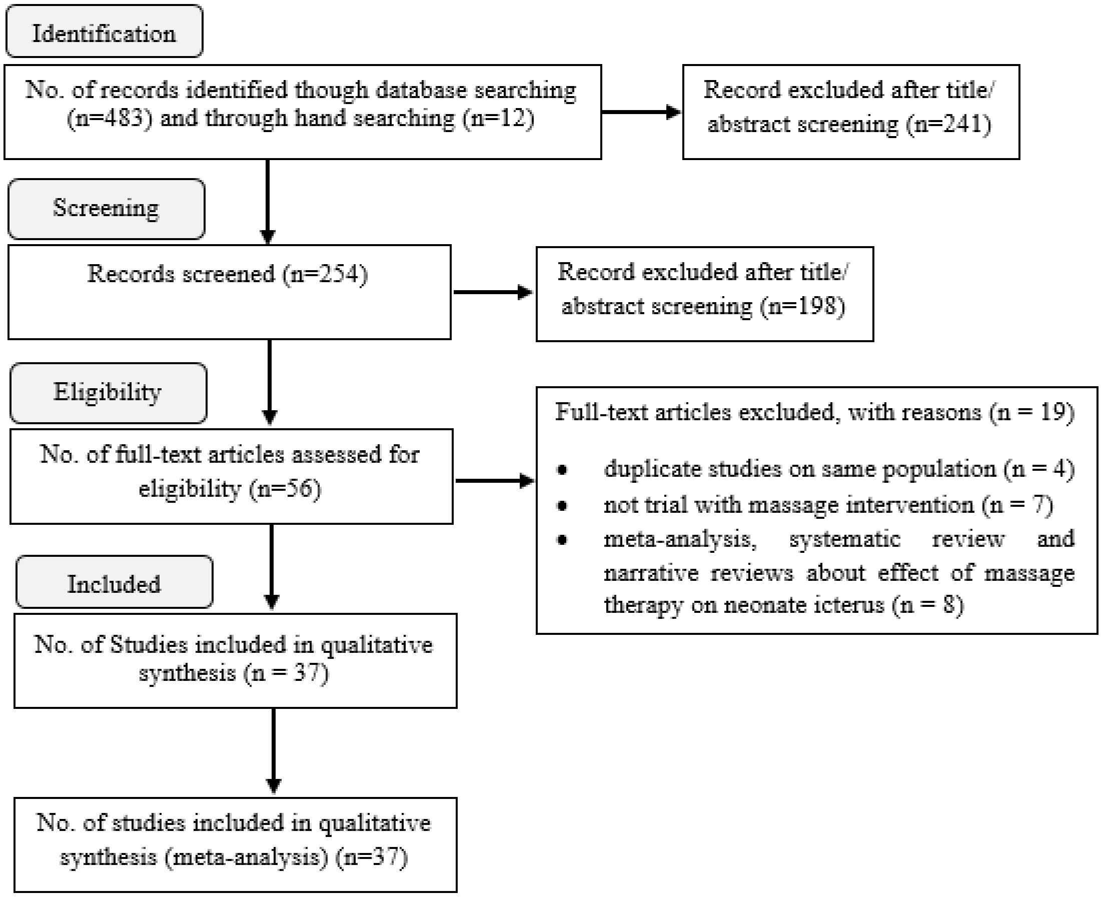

Figure 1. Flow chart of systematic literature search for trials, published through Juan 28, 2024, that met the study inclusion and exclusion criteria.

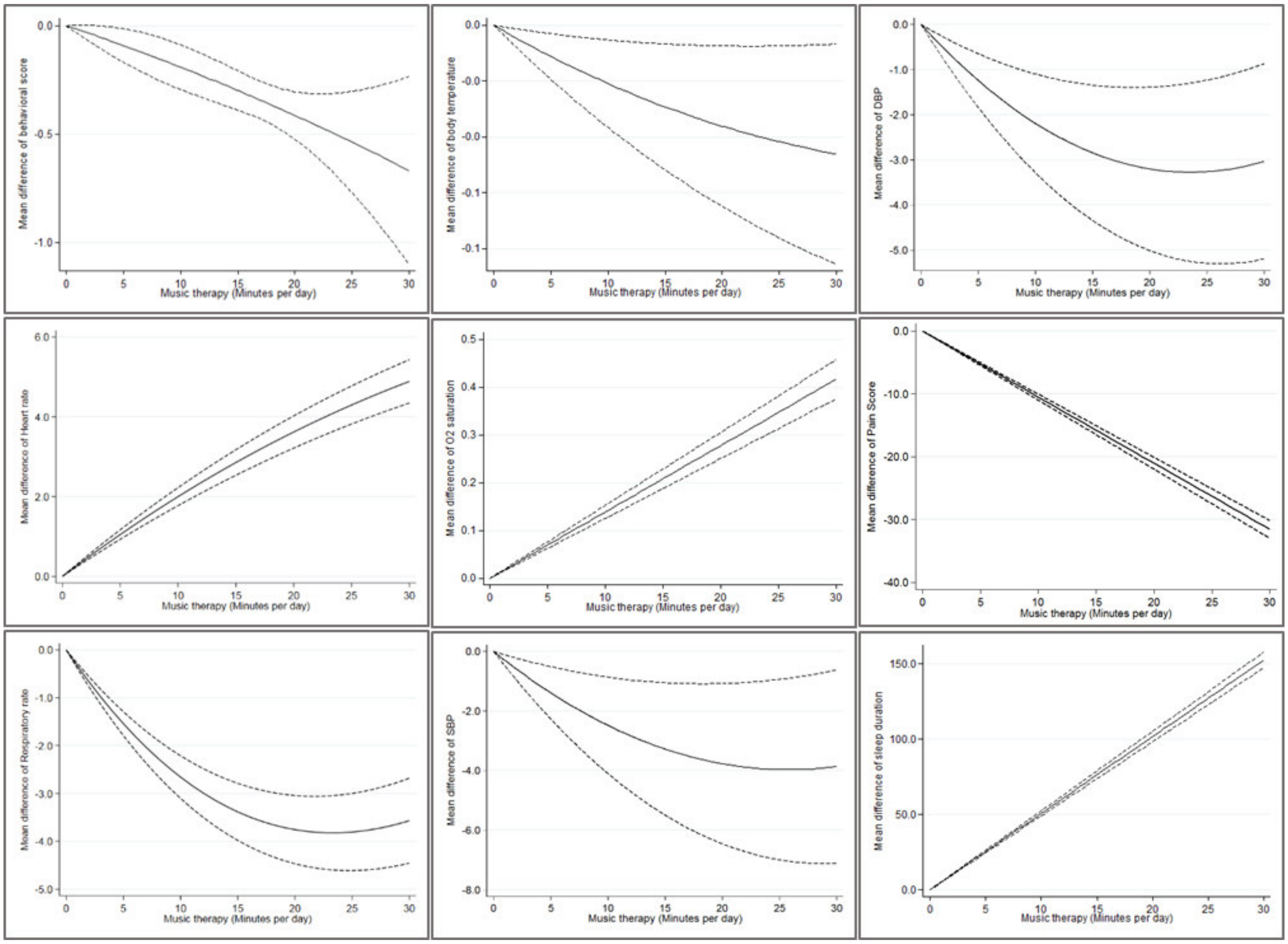

Figure 2. Dose-response meta-analysis between music therapy and neonatal outcomes in a random-effects dose-response model. The solid line indicates the mean difference, and long dashed lines indicate its 95% confidence interval.

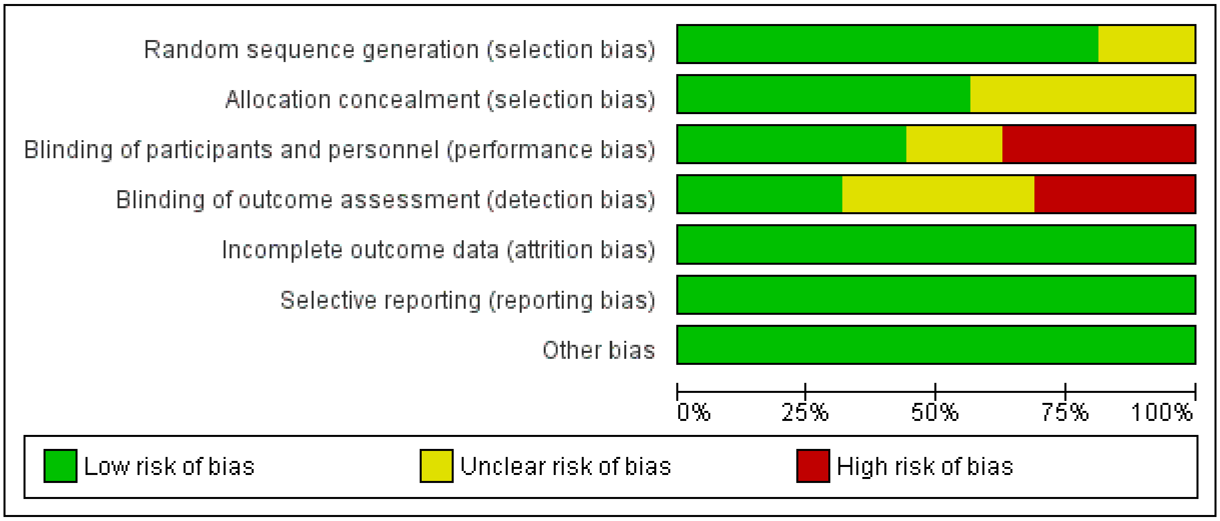

Figure 3. Review the author's judgment about each risk of bias item as percentages across all included studies using Cochrane Collaboration's Risk of Bias Tool.

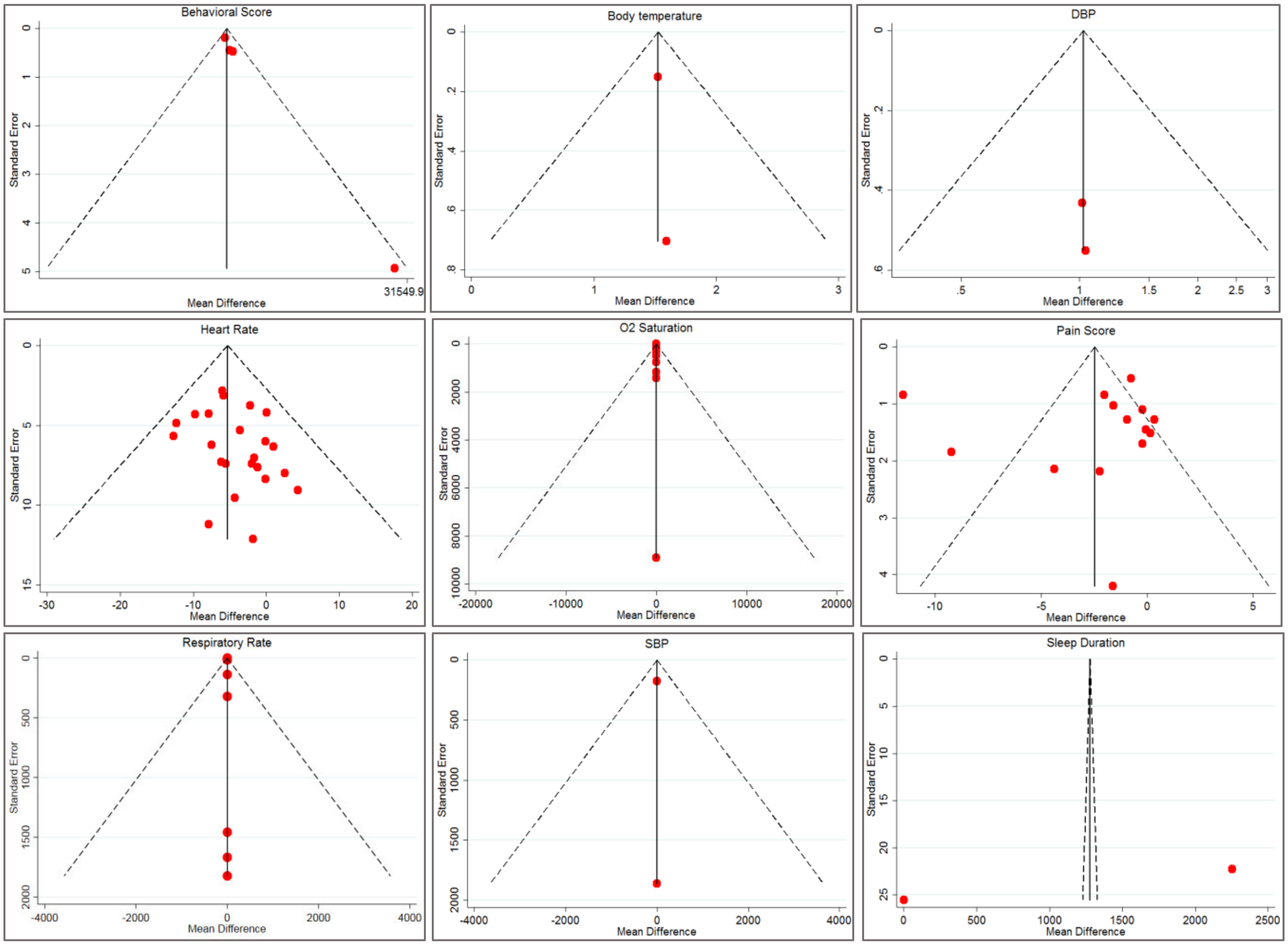

Figure 4. Funnel plots of included studies by neonatal outcomes.

Table1. Description of eligible studies reporting the effect of music therapy on neonatal outcomes.

First author, year[*]	country	Sample size (I/C)[§]	Outcomes	Type of music	Gestational age	Intervention characteristics
Alemdar, 2017 (32)	Turkey	34/68	Pain score, SaO2[¶]	Intrauterine sounds	premature infants with gestational age 28-36 weeks	1 day, one time in each day, 30 minutes in each time.
Alipour, 2013 (29)	Iran	30/60	Heart rate, Respiratory rate, SaO2, Behavioral Score	Lullaby music	premature infants with gestational age 28-37 weeks	2 days, one time each day, 10 minutes each time.
Arnon, 2006 (33)	Israel	31/31	Heart rate, Respiratory rate, SaO2, Behavioral Score[¥]	Live music	premature infants with gestational age 32-38 weeks	2 days, one time in each day, 30 minutes in each time.
Barandouzi, 2020 (34)	Iran	33/87	Pain score	Braham's lullaby music	premature infants with gestational age 32-35 weeks	3 days, one time in each day, 30 minutes in each time.
Caparros, 2018 (35)	Spain	11/11	Heart rate, Systolic and diastolic blood pressure, Respiratory rate, SaO2	Relaxing music	premature infants with gestational age 32-36 weeks	8 days, one time each day, 20 minutes each time.
Dehghani, 2015 (36)	Iran	27/26	Heart rate, Respiratory rate, SaO2, Body temperature	Music therapy is only mentioned, no details are given.	premature infants with gestational age 32-38 weeks	5 days, one time each day, 60 minutes each time.
Döra, 2021 (37)	Turkey	22/44	Heart rate, Respiratory rate, SaO2, Pain score	White noise and lullabies	premature infants with gestational age 32-36 weeks	2 days, one time each day, 2 minutes each time.
Howard, 2019 (38)	Ireland	10/11	Pain score, Respiratory rate, SaO2	Braham's lullaby music	premature infants with gestational age 28-32 weeks	2 days, one time each day, 10 minutes each time.
R. Keith, 2009 (39)	USA	22/22	Heart rate, Respiratory rate, SaO2	Schwartz music	premature infants with gestational age 32–38 weeks	1 day, one time each day, 18 minutes each time.
Kobus, 2021 (40)	Germany	20/20	Heart rate, Respiratory rate, SaO2	Individual singing, improvised singing by the music therapist and the use of the instrument sensual	premature infants with gestational age 28-32 weeks	11 days, one time each day, 50 minutes each time.
Kurdahi Badr, 2016 (41)	Lebanon	42/84	Heart rate, Respiratory rate, SaO2, Pain Score	Lullaby music or the music mothers listen to in the last trimester of pregnancy	premature infants with gestational age 28-36 weeks	3 days, one time in each day, 10 minutes in each time.
Namjoo, 2021 (42)	Iran	30/60	Heart rate, SaO2, Sleep duration	Mother's live lullaby and recorded lullaby	premature infants with gestational age 28-36 weeks	5 days, one time each day, 20 minutes each time.

Qolizadeh, 2018 (43)	Iran	32/32	Heart rate, Respiratory rate, SaO2, Body temperature	Holy Quran	Unclear	2 days, one time in each day, 30 minutes in each time.
Ranger, 2018 (30)	Germany	10/10	Heart rate, Respiratory rate, Pain score	Live music (The music intervention was performed by a psychologist who had studied hand harp music play in the NICU.)	premature infants with gestational age 28-36 weeks	2 days, one time in each day, 15 minutes in each time.
Uematsu, 2018 (44)	Japan	15/13	Heart rate, SaO2, Pain score	Braham's lullaby music	premature infants with gestational age 28-35 weeks	10 days, one time in each day, 30 minutes in each time.
Tandoi, 2014 (45)	Italy	17/17	Heart rate, Respiratory rate, SaO2, Pain score, Behavioral Score $^{\delta}$	The original sound includes an original track composed of different sounds such as fetal heartbeat, breathing, blood flow, and ambience sounds	premature infants with gestational age 32–37 weeks	3 days, three times in each day, 20 minutes in each time.
Amini, 2013 (46)	Iran	25/25	Heart rate, Respiratory rate, SaO2	Lullaby or the music mothers listen and classical music including Mozart	premature infants with gestational age 28-36 weeks	6 days, one time in each day, 20 minutes in each time.
Arnon, 2014 (47)	Israel	43/43	Heart rate, Respiratory rate, SaO2, Behavioral Score	Lullaby or maternal singing	premature infants with gestational age 32-36 weeks	2 days, one time in each day, 20 minutes in each time.
Aydin, 2012 (48)	Turkey	13/13	Heart rate, Respiratory rate, SaO2, Behavioral Score (stress score)	Classical music	Unclear	Once a day during the entire NICU hospitalization, 1 hour in each time.
Dur, 2022 (49)	Turkey	30/30	Heart rate, SaO2, Pain Score	Classical music including Mozart	premature infants with gestational age 28-36 weeks	1 day, one time each day, 1 minute each time.
Garunksteine, 2014 (50)	Lithuania	35/35	Heart rate, SaO2, Behavioral Score	Live & recorded lullabies	premature infants with gestational age 28-32 weeks	1 day, one time each day, 20 minute each time.
Jabraieili, 2016 (51)	Iran	25/25	SaO2	Brahms lullaby	premature infants with gestational age 29-34 weeks	3 sessions, 15 minute each session.
Lubetzky, 2010 (52)	Israel	5/13	resting energy expenditure	Classical music including Mozart	premature infants with gestational age 30-37 weeks	2 days, one time in each day, 30 minutes in each time.
Olischar, 2011 (53)	Australia	10/10	Sleep duration	Brahms lullaby	premature infants with gestational age ≥ 32 weeks	1 day, one time each day, 20 minute each time.

Rafael, 2017 (35)	Spain	11/11	Heart rate, Respiratory rate, SaO2, Systolic and diastolic blood pressure	A relaxing tune synthesized by Melodic computer system with no human intervention and following the recommendations of 10 expert musicians	premature infants with gestational age 32-36 weeks	3 days, three times in each day, 20 minutes in each time.
Schlez, 2011 (54)	Israel	52/52	Heart rate, Respiratory rate, SaO2, Behavioral Score	Live harp music	premature infants with gestational age 32-37 weeks	1 day, one time each day, 30 minute each time.
Standley, 2003 (55)	USA	16/16	Feeding rate	Recorded lullabies sung by female vocalists	Unclear	1 day, one time each day, 15 minute each time.
Standley, 2010 (56)	USA	34/34	Feeding rate	Recorded traditional lullabies sung by young female	premature infants with gestational age 32-36 weeks	1 day, one time each day, 15 minute each time.
Taheri, 2016 (57)	Iran	26/26	Heart rate, SaO2,	Recorded male lullaby	premature infants with gestational age 29-38 weeks	3 days, one time in each day, 40 minutes in each time.
Tang, 2018 (58)	China	30/30	Heart rate, SaO2, pain score	Lullabies & nursery rhymes, and more than 10 pieces of children's music with slower tempo that selected from “Chinese children music library”	premature infants with gestational age 28-36 weeks	1 day, one time each day, from 10 minutes before peripherally inserted central catheter puncture and continued until 10 minutes after operation completion.
Wipple, 2008 (59)	USA	14/13	Heart rate, Respiratory rate, SaO2	Lullaby music	premature infants with gestational age 32-37 weeks	Unclear but reported for before and during procedure
Lai, 2006 (60)	Taiwan	15/15	Heart rate, Respiratory rate, SaO2	Instrumental lullaby and aboriginal Taiwanese lullaby	premature infants with gestational age < 37 weeks	3 days, one time in each day, 60 minutes in each time.
Midilli, 2022 (61)	Turkey	20/19	Pain score	Braham’s lullaby music	premature infants with gestational age 35-41 weeks	3 days, one time in each day, 1 minutes in each time.
Mirzaei, 2022 (62)	Iran	18/18	Heart rate, SaO2	Piano lullaby and Gary Stadler	premature infants with gestational age < 36 weeks	1 day, one time each day, 5 minute each time.
Shah, 2017 (63)	Australia	35/35	Heart rate, SaO2, pain score	The “Deep Sleep” track from “Bedtime Mozart: Classical	premature infants with gestational age > 32 weeks	1 day, one time each day, 27 minute each time.

				Lullabies for Babies”		
Shukla, 2018 (64)	India	50/50	Pain score	Instrumental Indian classical flute music	premature infants with gestational age 28-36 weeks	1 day, one time each day, 10 minute each time.
Yarahmadi, 2024 (65)	Iran	40/40	Heart rate, SaO2, pain score	Intrauterine sounds	premature infants with gestational age 28-36 weeks	1 day, one time each day, 12 minute each time.

*: All trials were conducted in both sexes.

¶: Oxygen saturation.

§: I (sample size in intervention group/music therapy arm); C (sample size in control group).

¥: Behavioral states graded on the behavioural state instrument (BSI). Six different behavioural states were distinguished: State 1 quiet sleep, State 2 active sleep, State 3 drowsy, State 4 quiet awake, State 5 active awake and State 6 crying.

δ: The behaviour score in this trial meant the “calming/relaxing” effects of music (more details not available in the method of the article).

Click here to access/download
Supporting Information
PRISMA_2020_checklist.docx

Click here to access/download

Supporting Information - Compressed/ZIP File Archive

Minimal dataset.rar
