# Supplementary material for: Effect of music therapy on behavioral and physiological neonatal outcomes: A systematic review and dose-response meta-analysis
Source: PLoS One. 2025 Jan 8;20(1):e0316674. doi: 10.1371/journal.pone.0316674 (PMC11709260; doi:10.1371/journal.pone.0316674)
Supplement: Attachment — Submitted filename: review.docx [file pone.0316674.s005.docx]

**Reviewer comments**

1. The results in abstract needs to convey more clarity.

2. “If the full text of the paper was unavailable, we received it from Sci-Hub, free paper, Research Gate, or Send an email to the corresponding author”. This line should be removed.

3. Kindly clearly clarify the primary and secondary outcomes.

4. Kindly revisit the title since it does not convey exact meaning i.e. physiological parameters are not adverse outcomes.

5. Kindly attach the filled PRISMA checklist.

6. Kindly use the PRISMA flow diagram template and redo the study flow diagram.

7. Publication bias: funnel plot should be generated and attached.

8. search strategy use may miss few of the RCTs since it advisable to screen the article manually rather than relying on the search strategy.

9. Explain the need for this meta-analysis in introduction section.

10. The search strategy gave me 306 results on pubmed itself. So I believe there are more article that can be found and made useful and increase the comprehensiveness of the article.

11. I think the statistics can be made useful if time is translated to days or hours. Giving the fractional numbers may not appeal as applicable.

12. How were the confounders accounted to highlight true results of the music therapy?

--------------------------------------------------------------------------------------------------------------------------------------

1. The study presents the results of original research.

Yes

2. Results reported have not been published elsewhere.

Not sure

3. Experiments, statistics, and other analyses are performed to a high technical standard and are described in sufficient detail.

Needs lot of improvement.

4. Conclusions are presented in an appropriate fashion and are supported by the data.

It needs to be reframed to convey correct meaning. The results done mean improvement but change in the physiological parameters.

5. The article is presented in an intelligible fashion and is written in standard English.

Yes

6. The research meets all applicable standards for the ethics of experimentation and research integrity.

Yes

7. The article adheres to appropriate reporting guidelines and community standards for data availability.

Not sure. They should attach PRISMA checklist.
