# Supplementary material for: Effect of music therapy on behavioral and physiological neonatal outcomes: A systematic review and dose-response meta-analysis
Source: PLoS One. 2025 Jan 8;20(1):e0316674. doi: 10.1371/journal.pone.0316674 (PMC11709260; doi:10.1371/journal.pone.0316674)
Supplement: Attachment — Submitted filename: Response to Reviewers.docx [file pone.0316674.s006.docx]

Reviewer #1: Reviewer comments

1. Comment: The results in abstract needs to convey more clarity. Response: We rewrite result in abstract.
2. Comment: “If the full text of the paper was unavailable, we received it from Sci-Hub, free paper, Research Gate, or Send an email to the corresponding author”. This line should be removed. Response: We have deleted this phrase according to your opinion.
3. Comment: Kindly clearly clarify the primary and secondary outcomes. Response: Dear reviewer, we have re-written the first paragraph of the method in more detail according to PICOS criteria. In the outcome subsection, we mentioned the primary and secondary outcomes.
4. Comment: Kindly revisit the title since it does not convey exact meaning i.e. physiological parameters are not adverse outcomes. Response: Many thanks for your thoughtfulness. We changed the title to " Effect of music therapy on behavioral and physiological neonatal outcomes: A systematic review and Dose-Response Meta-analysis".
5. Comment: Kindly attach the filled PRISMA checklist. Response: Dear Professor Anurag Fursule, we have attached the completed PRISMA checklist.
6. Comment: Kindly use the PRISMA flow diagram template and redo the study flow diagram. Response: Many thanks for your attention. We added PRISMA flow diagram template in figure 1.
7. Comment: Publication bias: funnel plot should be generated and attached. Response: A funnel plot by neonatal outcomes was added to the result (Figure 3).
8. Comment: Search strategy use may miss few of the RCTs since it advisable to screen the article manually rather than relying on the search strategy. Response: We updated our search strategy and also used manual search. With this approach, 44 articles were finally included in our meta-analysis.
9. Comment: Explain the need for this meta-analysis in introduction section. Response: We are very grateful for your comment because it made us completely rewrite the introduction and write it more carefully and powerfully.
10. Comment: The search strategy gave me 306 results on PubMed itself. So I believe there are more article that can be found and made useful and increase the comprehensiveness of the article. Response: You enriched our article with your valuable comment. We updated our search strategy and also used manual search. With this approach, 44 articles were finally included in our meta-analysis.
11. Comment: I think the statistics can be made useful if time is translated to days or hours. Giving the fractional numbers may not appeal as applicable. Response: Dear reviewer, considering that in most studies, the entire period of inversion is less than 1 hour, minutes have a better meaning than hours. Using days instead of minutes is not very interesting. Because there may be two studies in which the length of the intervention period is 3 days in both of them. But the first one should play music for 20 minutes a day and the second one should play music for 5 minutes a day.
12. Comment: How were the confounders accounted to highlight true results of the music therapy? Response: The articles included in this meta-analysis were randomized clinical trials. According to epidemiology textbooks (such as Gordis epidemiology, chapter 10, pages 197-215 & Epidemiology beyond the basics, chapter 5, pages 175-202), randomization in clinical trial studies makes the compared groups balanced in terms of known and unknown confounders. In other words, randomization is a method of controlling confounding variables in clinical trials. Thank you for your valuable comment. Since I thought that the same concern might arise for the readers, I added a paragraph with the subtitle of confounding control in the method and explained this issue.

Reviewer #2: Comment: The authors made a great effort by using dose response meta-analysis by comparing to establish the link between music therapy and neonatal outcomes of O2 saturation, heart rate, respiratory rate, sleep duration, body temperature, and systolic and diastolic pressure. This dose-response meta-analysis has provided a reinforced cause and effect relationship between music and neonatal outcomes based on the observation of studies conducted in the past years. Response: Dear reviewer, thank you for your attention to our article.
