# Supplementary material for: Effect of music therapy on behavioral and physiological neonatal outcomes: A systematic review and dose-response meta-analysis
Source: PLoS One. 2025 Jan 8;20(1):e0316674. doi: 10.1371/journal.pone.0316674 (PMC11709260; doi:10.1371/journal.pone.0316674)
Supplement: Attachment — Submitted filename: Response to Reviewers.docx [file pone.0316674.s008.docx]

Comment 1: The discussion part requires enhanced clarity. Please examine the composition and sequence of sentences, especially in that section. Reply: Dear referee, thank you for your valuable comment. We have rewritten the discussion and highlighted it in yellow.

Comment 2: Please review Table 1, which contains the research studies included in your systematic review as well as the associated statistics. Table 1 lacks at least one of the articles cited in the reference list. Reply: Thank you for your high accuracy. Rafael's article is the same as Caparros 's article, so we removed it from Table 1. Corresponding to this deletion, other parts of the article were corrected.
